# Inferences regarding oneself and others in the human brain

**DOI:** 10.1371/journal.pbio.3001662

**Published:** 2022-05-23

**Authors:** Shinsuke Suzuki

**Affiliations:** Brain, Mind and Markets Laboratory, Department of Finance, Faculty of Business and Economics, The University of Melbourne, Parkville, Australia

## Abstract

The human brain can infer one’s own and other individuals’ mental states through metacognition and mentalizing, respectively. This Primer explores a new study in PLOS Biology that implicates distinct brain regions of the medial prefrontal cortex in metacognition and mentalizing.

## Teaser

The human brain can infer one’s own and other individuals’ mental states through metacognition and mentalizing, respectively. A new study in *PLOS Biology* has implicated distinct brain regions of the medial prefrontal cortex (PFC) in metacognition and mentalizing.

Are you confident about your decisions? In our daily lives, we often think about ourselves. The ability to internally evaluate an individual’s own mental state, including confidence, is called metacognition [1]. The development of confidence in one’s own decision is considered a crucial function of metacognition. The computational view posits that subjective confidence reflects the Bayesian posterior probability of the decision being correct [1]. Do you think your friend is confident about her/his decisions? In a social milieu, we often infer another individual’s confidence. Such inferences about others’ confidence are of particular importance in decision-making involving other individuals [2]. The ability to infer other individuals’ mental states, including confidence, is known as mentalizing [3].

Converging evidence from human neuroimaging studies, using functional magnetic resonance imaging (fMRI), implicate brain regions of the medial prefrontal cortex (PFC) in metacognition and mentalizing. Subjective confidence in perceptual decision-making is encoded in the medial PFC, decoupled from external cues about the environment pertaining to the decision (e.g., reliability of sensory evidence) [4]. A similar region in the medial PFC is also known to be recruited in tasks that require inferences about others’ beliefs decoupled from reality [5,6]. Despite the aforementioned findings, it remains unclear whether metacognition and mentalizing are associated with the common or distinct brain regions. To date, no studies have directly compared the neural mechanisms underlying these two cognitive processes.

**Fig 1:**
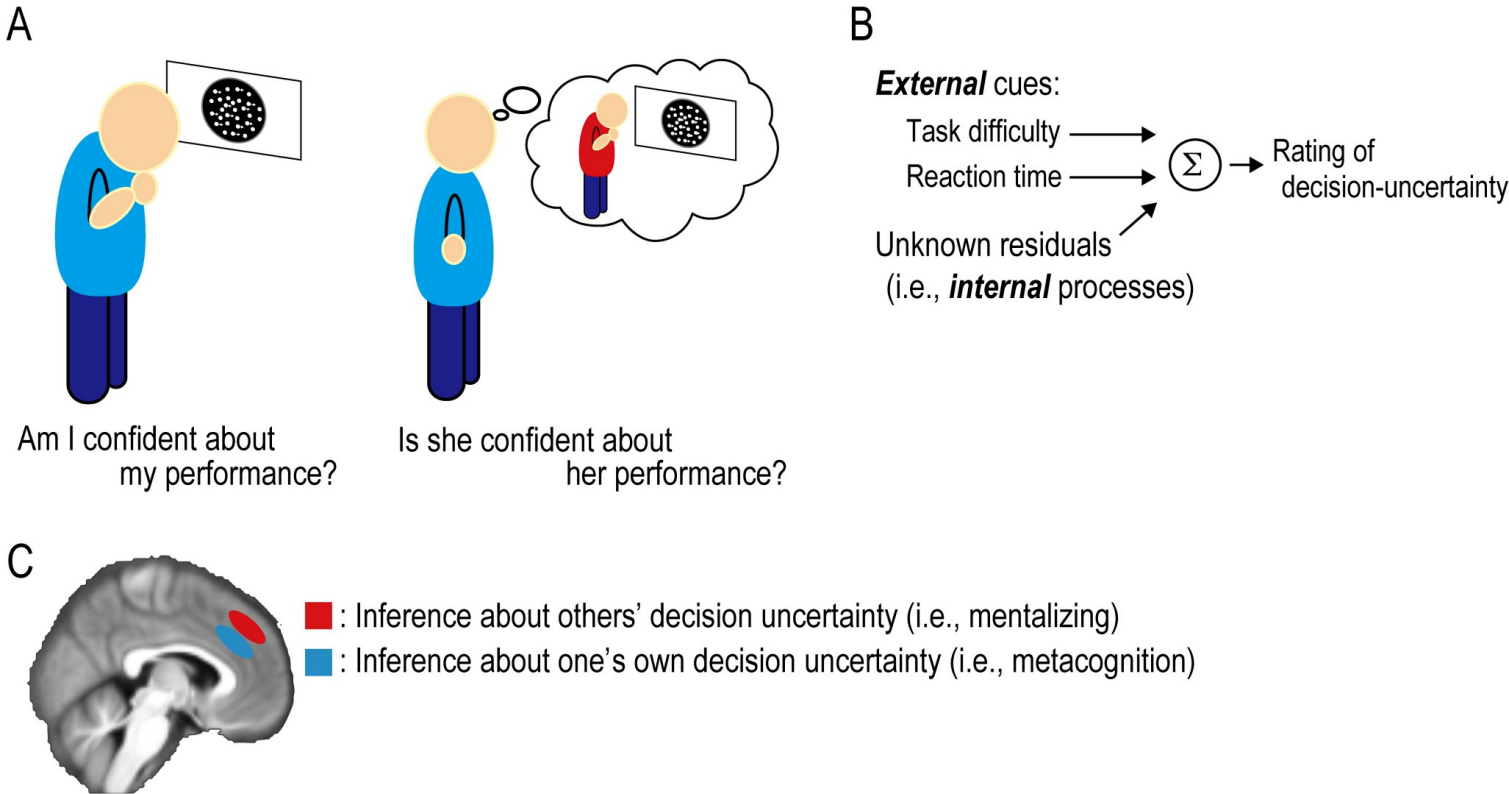
Summary of the experimental paradigm and main results. (A) In the metacognition experiment (left), participants themselves perform perceptual decision-making and rate the decision uncertainty (i.e., the inverted confidence). In the mentalizing experiment (right), they observe an unfamiliar individual performing the same perceptual decision-making and rate the other’s decision uncertainty. (B) In the data analysis, the authors reason that the rating of decision uncertainty reflects two types of information: external cues (i.e., the task difficulty and the reaction time) and internal mental process (i.e., the residuals that cannot be explained by effects of the external cues). Note that the exact content of the internal process remains unclear. (C) Distinct regions in the medial PFC are associated with metacognition (dACC in blue) and mentalizing (dmPFC in red). dACC, dorsal anterior cingulate cortex; dmPFC, dorsomedial PFC; PFC, prefrontal cortex.

The new work by Jiang and colleagues on this issue of *PLOS Biology* [7] is an important step toward a direct comparison of the neural signatures between metacognition and mentalizing. They devised a novel experimental paradigm to identify brain regions tracking “decision uncertainty” attributed to self (i.e., metacognition) and others (i.e., mentalizing). Notably, decision uncertainty in this study is referred to as the inverted version of confidence, reflecting a subjective sense of the decision away from being correct. In the metacognition experiment, participants performed a perceptual decision-making task [8] and rated the uncertainty surrounding their preceding decision (Fig 1A, left) while being scanned using fMRI. In the mentalizing experiment, they observed an unknown individual performing the same task and estimated the person’s decision uncertainty (i.e., the other’s belief about whether her/his own decisions were incorrect) (Fig 1A, right).

How do we estimate the decision uncertainty of another individual? The authors reasoned that the estimation relied on two external cues: task difficulty and reaction time (Fig 1B). In other words, the higher the task difficulty and the longer the reaction time, the higher the decision uncertainty of the individual (i.e., the lower the confidence). The behavioral data suggested that these external cues indeed predicted participants’ ratings of the decision uncertainty to some degree in the metacognition and mentalizing tasks; but at the same time, the prediction performance was found to be not so good. The findings imply the contributions of external cues, as well as unknown internal mental processes (i.e., the residuals that cannot be attributed to the effects of external cues), to metacognition and mentalizing (Fig 1B).

Additionally, the authors aimed to identify brain regions that track external environmental cues and internal mental processes for metacognition and mentalizing. Notably, in this study, as a proxy for the internal process, they focused on the residuals after regressing out the external cue information from the ratings of decision uncertainty (Fig 1B). This is based on the assumption that the estimated residuals, at least in part, reflect an internal mental process that cannot be attributed to external cues. They found that distinct regions in the medial PFC were associated with metacognition and mentalizing (Fig 1C). Specifically, the dorsal anterior cingulate cortex (dACC) encodes the internal mental process for inference of one’s own decision uncertainty in the metacognition task, and the dorsomedial PFC (dmPFC) encodes the internal process for inference of others’ decision uncertainty in the mentalizing task. Conversely, external cues such as task difficulty and reaction time were found to be represented in the inferior parietal lobe.

One of the most significant contributions of this study is the characterization of the role of the medial PFC in metacognition and mentalizing. The medial PFC is purportedly involved in representing one’s own decision confidence (i.e., metacognition) [4] and inferring others’ mental states (i.e., mentalizing) [5]. This study delineates the functional dissociation of the two subregions in the medial PFC: The dACC represents the internal mental process for inference of one’s own decision uncertainty, and the dmPFC represents the internal process for inference of anonymous others’ decision uncertainty.

Some issues should be addressed in future studies. One may wonder what is the exact content of the internal mental process. This question could not be explicitly answered in this work as the internal process was characterized by the “residuals” after regressing out the external cue information from the ratings of decision uncertainty (Fig 1B). An interesting avenue for future research is to open a black box. Moreover, higher-order cognitions such as metacognition and mentalizing are unlikely to be implemented in a single brain region. It would therefore be interesting to uncover how networks of brain regions causally underlie metacognition and mentalizing. This can be accomplished by combining functional neuroimaging and brain stimulation [9].

Are inferences about oneself and others similar to each other? This has been a long-standing question in psychology and philosophy [10]. This study provides an essential scaffold for addressing this issue from the perspective of the underlying neural mechanisms.

## Abbreviations:

dACC: dorsal anterior cingulate cortex
dmPFC: dorsomedial PFC
fMRI: functional magnetic resonance imaging
PFC: prefrontal cortex
